# Randomized prospective trial to detect and distinguish between medication nonadherence, drug-drug interactions, and disease progression in chronic cardiometabolic disease

**DOI:** 10.1186/s12875-023-02042-4

**Published:** 2023-04-15

**Authors:** John W Peabody, Divya Ganesan, Czarlota Valdenor, David Paculdo, Joshua Schrecker, Christopher Westerfield, Rebecca Heltsley

**Affiliations:** 1 QURE Healthcare, San Francisco, CA USA; 2 Aegis Sciences Corporation, Nashville, TN USA; 3 University of California, San Francisco, CA USA; 4 University of California, Los Angeles, CA USA; 5 450 Pacific Avenue, Suite 200, San Francisco, CA 94133, 415-321-3388 USA

**Keywords:** Adverse drug reactions, Cardiometabolic diseases, Disease progression, Drug-drug interactions, Medication nonadherence, Polypharmacy

## Abstract

**Background:**

Disentangling nonadherence (NA), drug-drug interactions (DDIs), and disease progression from each other is an important clinical challenge for providers caring for patients with cardiometabolic diseases. NAs and DDIs are both ubiquitous and often overlooked. We studied a novel chronic disease management (CDM) test to detect medication adherence and the presence and severity of DDIs.

**Materials and methods:**

We conducted a prospective, randomized controlled trial of 236 primary care physicians using computer-based, simulated patients, measuring clinical care with and without access to the CDM test. The primary outcomes were whether use of the CDM test increased the accuracy of diagnoses and ordering better treatments and how effective the intervention materials were in getting participants to order the CDM test.

**Results:**

Physicians given the CDM test results showed a + 13.2% improvement in their diagnosis and treatment quality-of-care scores (p < 0.001) in the NA patient cases and a + 13.6% improvement in the DDI cases (p < 0.001). The difference-in-difference calculations between the intervention and control groups were + 10.4% for NA and + 10.8% for DDI (p < 0.01 for both). After controlling for physician and practice co-factors, intervention, compared to control, was 50.4x more likely to recognize medication NA and 3.3x more likely to correctly treat it. Intervention was 26.9x more likely to identify the DDI and 15.7x more likely to stop/switch the interacting medication compared to control. We found no significant improvements for the disease progression patient cases.

**Conclusion:**

Distinguishing between nonadherence, drug-drug interactions, and disease progression is greatly improved using a reliable test, like the CDM test; improved diagnostic accuracy and treatment has the potential to improve patient quality of life, medication safety, clinical outcomes, and efficiency of health delivery.

**Trial Registration:**

clinicaltrials.gov (NCT05192590).

**Supplementary Information:**

The online version contains supplementary material available at 10.1186/s12875-023-02042-4.

## Background

Treating cardiometabolic diseases relies on sustained efforts ranging from lifestyle modification, public health measures, and procedural interventions to achieve better health outcomes [1]. The mainstay of cardiometabolic treatment and secondary prevention, however, is poly-pharmacologic therapy, with the majority of cardiometabolic patients on 7–15 different medications [2].

The physician, already challenged to prescribe the appropriate medication at the correct dose, must also hope that patients take their prescribed medication. Numerous studies indicate > 40% of patients with chronic conditions do not correctly follow their prescribed drug regimens, which is due to many factors including the sheer volume of medications they are prescribed [3–6]. With this level of polypharmacy, patients are at increased risk of medication nonadherence (NA) and for drug-drug interactions (DDIs) [7]. Researchers found the majority of the drugs implicated in DDIs are prescribed for cardiometabolic diseases [7].

Adding to the challenge, physicians and other providers must not only disentangle nonadherence from DDIs but also separate these two issues from disease progression [5]. Moreover, the busy physician must sort through these three possibilities in a dynamic, time-constrained clinical setting [8]. Unsurprisingly, nonadherence and DDIs are overlooked at best and, at worse, attributed to disease progression [9].

An important reason physicians have difficulty in distinguishing nonadherence from DDIs and disease progression is lack of an effective and standardized method to test for these conditions [10]. The current measures to assess for nonadherence have all proved wanting [11]. For example, point-of-care drug alerts have the potential to prevent DDIs, but have had limited success, with as many as 98% of these alerts overridden [12]. Ideally, an objective measurement of adherence would be made even more useful if it could also test for relevant DDIs in polypharmacy patients.

Prior to this study, scientists at Aegis Sciences Corporation have developed a Chronic Disease Management (CDM) test that detects medication adherence and the presence and severity of DDIs. The CDM test detects ingested substances from an oral fluid sample and tests for 150 of the most common cardiometabolic medications, including angiotensin-converting enzyme inhibitors, angiotensin receptor blockers, beta blockers, diuretics, statins, sulfonylureas, antithrombotic agents, and vasodilators. The CDM test provides the physician with qualitative adherence data on these medications and also identifies potential DDIs. Through Aegis, we were allowed use of simulated CDM test results and funded to determine whether clinical practice change occurred.

We used simulated patients in a randomized controlled intervention trial (RCT) to evaluate whether simulated results from the CDM test changed primary care physician behavior and led to better, more accurate diagnoses and treatment among patients presenting with chronic cardiometabolic diseases and improved their ability to distinguish between medication nonadherence, DDIs, or disease progression.

## Methods

### Overview

Between October 2021 and February 2022, we conducted a prospective, cross-sectional RCT using simulated Clinical Performance and Value® (CPV®) vignettes. The study was conducted online and assessed the clinical utility of the CDM test among United States primary care physicians (PCPs) in the evaluation, work-up, diagnosis, and management of patients with chronic cardiometabolic disease with either NA, DDIs, or disease progression. We measured the participating physicians’ clinical care before and after introduction of the CDM test. Participants each cared for three simulated CPV® patients per two rounds, for a total of six patient cases.

### Ethics

This study was conducted in accordance with ethical standards, approved by the Advarra Institutional Review Board, Columbia, MD, USA, and listed in clinicaltrials.gov (NCT05192590, 14/01/2022). Voluntary, informed consent was obtained from all participants.

### Sample Size Calculation

From previous work, we know that a 5% change in CPV scores are both statistically and clinically significant. [13] We, therefore, calculated the sample size necessary to be able to detect a 5% difference in diagnosis and treatment (DxTx) at 80% power between intervention and control within one of the case variants (NA, DDI, or disease progression). With these assumptions and a standard deviation of 11%, the calculated sample size for each arm was 75.

### Physician Selection

We recruited and enrolled practicing PCPs from a national roster. The eligibility criteria were: (1) board-certified, (2) currently practicing in internal or family medicine between 2 and 35 years, (3) practicing in a community or non-academic setting, (4) caring for more than 40 patients weekly, (5) commonly treating patients with cardiometabolic conditions such as atrial fibrillation (Afib), coronary artery disease, heart failure (HF), diabetes (DM), and hypertension (HTN), (6) practicing in the United States, (7) English-speaking, and (8) able to access the internet. All eligible participants were randomly assigned using a coin flip methodology into one of three study arms. Recruitment continued until we reached at least the sample size as calculated above.

261 PCPs met the inclusion criteria. Of this total, 8 participants withdrew from the study and 17 participants failed to complete all six patient cases. 236 physicians completed 1,416 simulated patient cases. Of these 236, 76 were in control, 77 in intervention 1, and 83 in intervention 2. In the first round of patient cases, all physicians were naïve and without access to the CDM test results.

### Intervention

We provided identical educational materials consisting of a physician-targeted slide deck, a fact sheet detailing the science behind the test, the sample collection method, and a sample report to both intervention groups, who were required to view these materials before progressing to round 2 and completing the study. Two weeks after reviewing the educational materials, both intervention groups were asked to complete three new CPVs during round 2 of the study.

Intervention 1 were given the CDM test results as they completed the round 2 patient cases, and intervention 2 had the option of ordering the CDM test while caring for their round 2 patients. The simulated CDMT results could be viewed immediately after ordering and are available in a format that is identical to the actual CDMT result. Control arm physicians in round 2 had access to the current standard of care diagnostic tools but did not have access to the CDM test results.

### Data sources

We had two sources of data: the physician survey and the physicians’ responses to the CPVs.

### Physician survey

After physicians were enrolled into the study, they were asked to answer a brief questionnaire detailing their practice characteristics, their patient level and types, and their own demographic background. The survey included questions on employment status, location of practice, practice type, and patient make-up, among others.

### Clinical Performance and Value (CPV) vignettes

CPVs are a validated online patient simulation tool owned by QURE Healthcare which have been widely used to measure clinical care [13, 14]. Several authors, through their affiliation with QURE Healthcare, were authorized to use the CPV vignettes. These vignettes are open-ended questions simulating typical clinical encounters involving four domains of care: (1) history taking, (2) physical examination, (3) diagnostic workup, and (4) making a diagnosis and treatment (DxTx) plan including follow-on care.

With between 61 and 74 evidence-based criteria evaluated for each CPV, participant responses were scored by two trained expert physicians, working independently, using pre-determined criteria (see Supplement 1) based on current standards of care to measure individual physician care. In cases of disagreement, a third physician would adjudicate for the final score. All three physicians were blinded to the study arm assignment of the participant. We generated a quality-of-care percentage score based upon the number of responses matching the evidence-based criteria (range from 0 to 100%). Higher percentage scores indicated greater adherence to the evidence base in clinical care provided.

We note the DxTx score has proven helpful to understand the challenges clinicians face—DDI DxTx scores were 22.9% in our previous research indicating that both diagnosis and treatment of DDIs are significant clinical problems [15]. CPV cases have been used to evaluate and compare clinical practices of healthcare providers in a comprehensive range of clinical conditions and types of clinical practices [16–19].

### Chronic cardiometabolic disease vignettes

We constructed nine CPV vignettes on a 3 × 3 matrix with three patient case types and three variants (see Supplement 2). The case types included patients with Afib, HF, or DM/HTN. The three variants included patients who were not at their therapeutic goal because of: medication nonadherence but no DDI (NA); because of a DDI but adherent to their medications (DDI); and disease progression who were both adherent to their medications and had no DDI (AND). For the AND patients, a diagnosis of disease progression was made with either explicitly diagnosing worsening disease, selecting a new medication, increasing the dose of medication, or referring to a specialist. To avoid ordering effects, every participant cared for three CPV patients, one randomly assigned from each case type and from each variant type. At the end of the two rounds, each participant completed a total of six patient cases, three patients per round, one from each case type and case variant, with no provider seeing the same patient more than once.

### Study outcomes and analysis

The study sought to determine the clinical utility of the CDM test. Accordingly, the primary outcome is whether using CDM test improved patient care by increasing the diagnosis of NA or DDI *or* if it led to changes in medication treatment. More specifically, we wanted to: (1) determine the change in frequency of identifying/diagnosing NA, DDIs, and AND; (2) measure the difference in treating these three patient types as a result of receiving the CDM test reports; and (3) explore how effective the intervention materials were in getting participants to order the CDM test. All primary outcomes were based on a 0-100% scale, with primary analyses calculated as a difference-in-difference percentage between intervention and control and as an odds ratio with 95% confidence interval as determined by multiple variable logistic regression.

Secondary outcomes included the effect of provider and clinical practice characteristics on care, cost implications of using the CDM test, and identifying the best use cases of the CDM test. The effect of provider and clinical practice characteristics were determined by inserting these variables into the multiple variable regression models. Cost analysis was done by measuring differential rates of diagnostic ordering selected by each arm and multiplying by average Medicare reimbursement rates for these workups. Use case determination was made through difference-in-difference and logistic subanalyses of the clinical variants presented to the participants.

All analyses were done in Stata 14.2.

### Patient and Public Involvement

No patients involved.

## Results

### Physician Characteristics

236 board-certified PCPs met the eligibility requirements, completed the physician questionnaire and six CPV patient cases (Table 1). Over half of participants worked in a suburban setting and a quarter worked in solo private practice. Intervention arms had slightly more male participants (control: 68.4%; intervention 1: 77.9%; intervention 2: 75.9%; p = 0.367) and intervention 1 had more internal medicine physicians (control: 54.0%; intervention 1: 61.0%; intervention 2: 51.8%; p = 0.475).

**Table 1:** Physician Baseline Characteristics, by Study Arm

	Control	Intervention 1	Intervention 2	p-value
N	76	77	83	--
**Male**	68.4%	77.9%	75.9%	0.367
**Age**	56.1 + 8.7	57.0 + 7.8	56.3 + 8.2	0.940
**Internal Medicine**	54.0%	61.0%	51.8%	0.475
**Practice Type**				
Hospital-Based	9.2%	11.7%	12.1%	0.266
Private, Multi-Specialty	34.2%	32.5%	20.5%	
Private, Single Specialty	30.3%	29.9%	45.8%	
Private, Solo	26.3%	26.0%	21.7%	
Region*				
Northeast	33.3%	24.7%	28.9%	0.829
South	28.0%	32.5%	33.7%	
Midwest	21.3%	20.8%	22.9%	
West	17.3%	22.1%	14.5%	
Setting*				
Urban	28.0%	37.7%	25.3%	0.248
Suburban	61.3%	54.6%	57.8%	
Rural	10.7%	7.8%	16.9%	
Employed by Practice*	82.7%	77.9%	69.9%	0.157
**Payer, %**				
Medicare	37.7%	34.5%	36.5%	0.654
Medicaid	10.2%	8.7%	9.1%	0.571
Commercial	45.0%	49.7%	48.8%	0.823
Self	4.5%	5.0%	6.4%	0.252
Other	2.7%	2.0%	2.2%	0.730
Participant in CMS Quality Program*				
Yes	30.7%	31.2%	41.0%	0.356
No	52.0%	58.4%	44.6%	
Don’t know	17.3%	10.4%	14.5%	
**Medication Reconciliation Used**				
Pharmacy reconciliation	90.8%	89.6%	88.0%	0.843
EMR alert	85.5%	80.5%	75.9%	0.310
Self-report	67.1%	68.8%	73.5%	0.659
Urine drug screen	35.5%	32.5%	37.4%	0.809
Confirmation drug testing	19.7%	15.6%	25.3%	0.308
Digital pills	2.6%	1.3%	1.2%	0.744
None	0.0%	0.0%	2.4%	0.156

*One control participant missing data

### Diagnosis-and-Treatment Domain Scores

At baseline, we found wide variation in DxTx scores among participants caring for patients with cardiometabolic diseases. Across all patient cases, DxTx ranged from 0 to 75%, averaging 21.7%±13.4%. DxTx scores among the three case types were 22.7% for Afib, 22.4% for HF, and 19.9% for DM/HTN (p > 0.05). Breaking out the results by the three case variants, we found participants diagnosed and treated medication nonadherence 23.8%±13.4%, more frequently than DDI 17.7%±14.1%, and about the same as AND 23.5%±11.8%; these differences were significant (p < 0.001). There were no significant differences in DxTx scores between study arms at baseline (Table 2) (p > 0.05 for all case variants).

**Table 2:** Diagnosis-Treatment Scores by CPV Variant

**Medication Nonadherence**			
Study arm	R1	R2	p-value
Control	21.2%±12.1%	24.0%±14.6%	0.195
Intervention 1	25.0%±13.2%	38.2%±22.1%	< 0.001
Intervention 2	25.3%±14.4%	30.4%±20.0%	0.031
p-value	0.102	< 0.001	
**Drug-drug Interaction**			
Study arm	R1	R2	p-value
Control	16.1%±13.6%	18.8%±13.7%	0.216
Intervention 1	17.4%±15.1%	31.0%±18.4%	< 0.001
Intervention 2	19.4%±13.7%	19.7%±17.9%	0.449
p-value	0.331	< 0.001	
**Disease Progression**			
	R1	R2	p-value
Control	20.8%±10.9%	23.6%±10.9%	0.140
Intervention 1	24.9%±12.0%	20.1%±10.3%	0.004
Intervention 2	24.6%±11.4%	21.5%±10.3%	0.036
p-value	0.055	0.124	

We then compared control to the first intervention in a pre-post analysis. The formal difference-in-difference estimations using a fixed effects model showed a + 10.4% improvement in recognizing and treating NA and a + 10.8% improvement for identifying and treating DDI (p < 0.01 for both). There was no round over round improvement in the DxTx score for the AND patient cases.

After controlling for gender, internal medicine specialty, age, region, practice locale and type, the fixed-effects model showed practicing in the West (+ 4.3%, 95% C.I. +2.2% to + 6.4%) and in non-urban environments (+ 2.1%, 95% C.I. +0.3% to + 3.9%) were correlated with higher DxTx scores. Comparing intervention to control, we found intervention 1 providers performed significantly better than controls across all patient cases (+ 4.5%, 95% C.I. +0.6% to + 8.3%). By case variant, the intervention group improved significantly for both the NA (+ 10.8%, 95% C.I. +3.4% to + 18.2%) and DDI cases (+ 11.0%, C.I. +4.2% to + 17.9%), but not for the AND cases (-7.6%, 95% C.I. -12.6% to -2.6%).

By case type (Afib, HF, and DM/HTN), we found no significant improvement in DxTx score for the intervention group in the difference-in-difference fixed effects modeling (Afib: +2.9%; HF: +3.8%; DM/HTN: +6.7%; p > 0.05 for all). However, we did see improved identification of both NA and DDI across all case types (Afib, O.R. 39.7, 95% C.I. 5.1-309.4; HF, O.R. 19.2, 95% C.I. 1.6-230.7; DM/HTN, O.R. 99.0, 95% C.I. 8.8-1179.5).

### Identification and Treatment of Underlying Cause by Case Variant

We next disaggregated the combined DxTx score to explore how well physicians identified and managed the root problems of their patient’s symptoms (medication nonadherence, DDI, or disease progression).

#### Medication nonadherence

At baseline, among the NA patient cases, providers identified NA in their patients only 2.0% of the time, with no difference between study arms (p = 0.414) (Table 3a). After introduction of the CDM test, intervention increased their detection of nonadherence from 1.3 to 39.0% (p < 0.001), while control stayed nearly the same (2.6–2.7%, p = 0.989).

**Table 3:** Primary Variant Diagnosis and Related Treatment, by Case Variant

(a). Primary Variant Diagnosis								
**Nonadherence (Variant NA)**								
		R1		R2		p-value		
Control		2.6%		2.7%		0.989		
Intervention 1		1.3%		39.0%		< 0.001		
Intervention 2		4.8%		14.8%		0.031		
p-value		0.414		< 0.001				
**DDI (Variant DDI)**								
		R1		R2		p-value		
Control		9.2%		9.3%		0.979		
Intervention 1		6.5%		57.1%		< 0.001		
Intervention 2		9.6%		18.5%		0.102		
p-value		0.745		< 0.001				
**Other (apixaban resistance, HF progression, HTN-caused symptoms) (Variant AND)**								
		R1		R2		p-value		
Control		50.0%		72.0%		0.006		
Intervention 1		64.9%		57.1%		0.321		
Intervention 2		66.3%		58.0%		0.277		
p-value		0.071		0.105				
(b) CMD-related Primary Treatment								
**Continue stopped medication (Variant NA)**								
	R1		R2		p-value			
Control	6.6%		5.3%		0.747			
Intervention 1	14.3%		35.1%		0.003			
Intervention 2	9.6%		13.6%		0.430			
p-value	0.283		< 0.001					
**Stop/switch interacting substance (Variant DDI)**								
	R1		R2		p-value			
Control	32.9%		12.0%		0.002			
Intervention 1	32.5%		64.9%		< 0.001			
Intervention 2	39.8%		21.0%		0.009			
p-value	0.553		< 0.001					
**Other (shift med, advise HF progression, HTN workup) (Variant AND)**								
	R1		R2		p-value			
Control	14.5%		32.0%		0.011			
Intervention 1	23.4%		28.6%		0.462			
Intervention 2	25.3%		33.3%		0.258			
p-value	0.211		0.803					

The improvement in NA case diagnosis in intervention 1 led to improved clinical care manifested by continuing medication and discussing the importance of medication adherence. After introduction of the CDM test, intervention continued the nonadherence medications by an additional 20.8% (p = 0.003) (Table 3b). Control performance was unchanged at 6.6% and 5.3% round-to-round (p = 0.747).

Regression modeling confirmed that intervention were 50.4x more likely to identify NA (95% C.I. 2.9-871.2) and 3.3x more likely to continue the medications for which their patients were nonadherent, although the latter proved not to be significant (O.R. 0.6–19.3).

#### Drug-drug interactions

At baseline, identification of DDIs was modest in both control and intervention (7.8% for both arms, p = 0.532). After introduction of the CDM test, intervention significantly improved their ability to identify DDIs, increasing from 6.5 to 57.1% (p < 0.001) compared to contro which did not change (9.2–9.3%, p = 0.979).

After identifying more DDIs, intervention was nearly twice as likely to make a clinical adjustment by typically either stopping the interacting medications or shifting to a different medication (32.5–64.9%, p < 0.001) compared to control (32.9–12.0%, p = 0.002).

Here, the fixed effects model confirmed that intervention was 26.9x more likely to identify the DDI (95% C.I. 5.6-130.6) and 15.7x more likely to stop the interacting substance (95% C.I. 5.0–49.0).

#### Disease progression

Although making the diagnosis of disease progression trended in the right direction for these patient cases at + 3.2%— this trend did not reach statistical significance (p = 0.838) and, in the difference-in-difference estimation, the intervention group was 0.3x as likely to diagnose disease progression and 0.3x as likely to advance the medication regimen or increase medication dose.

### Intervention 2 Results

We wanted to determine whether the educational materials increased awareness of nonadherence or DDIs and if so, how this impacted practice and test ordering by physicians.

Overall, intervention 2 only ordered the CDM test in 12.4% of patient cases with no significant difference by case variant (p = 0.892). When given the option of ordering the CDM test after reviewing the education materials, they had a nonsignificant improvement in their DxTx domain scores for NA cases of + 2.0%, (p = 0.542) and no improvement in the other case variants when compared to baseline scores. When we looked at diagnosing NA, the second intervention improved from 4.8 to 14.8% (p = 0.031), while the control remained the same (p = 0.989). Intervention 2 also improved from 9.6 to 18.5% in diagnosing DDIs (p = 0.102). When we controlled for physician and practice characteristics, we found a 3.6 × (95% C.I. 0.3–34.8) improvement by intervention 2 to identify nonadherence in the NA patient cases and 1.9 × (95% C.I. 0.4–9.8) improvement in treatment compared to controls. Similarly, for the DDI cases, intervention 2 was 2.3 × (95% C.I. 0.5–9.4) more likely to identify the patient’s DDI and 1.5 × (95% C.I. 0.5–4.4) more likely to treat it.

We split intervention 2 into two subgroups and analyzed those who chose to order the CDM test (Int2A) and those who did not order the test (Int2B). At baseline, there was no difference between the two subgroups. However, after introducing the education materials, Int2A were significantly better in DxTx (43.2%±24.8% vs. 21.2%±14.0%), making the appropriate primary diagnosis (60.0% vs. 18.9%, p < 0.001), and in discontinuing the offending agent(s) (50.0% vs. 18.2%, p < 0.001) compared to Int2B.

Int2A had similar scores to intervention 1, and Int2B scored similarly to control (Table 4). Int2A, like intervention 1, were significantly more likely to improve DxTx scores in the NA (+ 32.4%) and DDI (+ 20.7%) patient cases but not in the AND cases (-2.4%). They were also significantly more likely to identify medication nonadherence (O.R. 41.6x) and DDI (O.R. 56.2x) but not disease progression (O.R. 0.3x). In treatment, Int2A were significantly more likely to stop the interacting medicine (O.R. 14.0x) in the DDI patient cases, and although improvement was seen, they were not significantly more likely to continue the stopped medication in the nonadherence cases (O.R. 7.2x).

**Table 4:** Intervention 2 Comparison by CDM Usage Versus Control, Multivariate Regression Modeling

	Intervention 2 w/CDM test	Intervention 2 w/o CDM test
**Change in DxTx Score, %**		
Medication nonadherence	+ 32.4%***	-4.0%
DDI	+ 20.7%**	-5.0%
Disease progression	-2.4	-6.4%
**Change in Primary Underlying Diagnosis, O.R.**		
Medication nonadherence	41.6x*	1.1x
DDI	56.2x**	1.1x
Disease progression	0.3x	0.3x
**Change in Primary Related Treatment, O.R.**		
Medication nonadherence	7.2x	0.7x
DDI	14.0x*	0.9x
Disease progression	0.3x	0.3x

* p < 0.05

** p < 0.01

*** p < 0.001

### Economic Changes in Diagnostic Ordering

When we examined the economic impact of the CDM test, we found that intervention 1 physicians ordered 0.3 fewer low-value tests per case (95% C.I. 0.0 to 0.6). This decrease in test ordering translates to a per case savings of $119 (95% C.I. $20 to $217).

## Discussion

In patients with chronic cardiometabolic diseases, healthcare outcomes depend upon correct diagnoses and effective treatment regimens [20]. Medication nonadherence and DDIs are underrecognized but significant barriers to effective medical treatment [21]. Our earlier study among US PCPs revealed that medication nonadherence and DDIs was recognized and diagnosed in just 3.6% and 8.9% of cases, respectively—despite 99% of participants indicating that they used some form of medication reconciliation in their everyday practice [22]. Treatment suffered, too: 24.4% of NA cases and 40.5% of DDI cases were inappropriately treated.

We conducted a RCT to determine if the test improved the recognition, diagnosis, and medication management of medication nonadherence and DDIs in patients with chronic cardiometabolic diseases. The results showed large differences between the intervention and the control groups: physicians who used the CDM test were 50.4x more likely to diagnose medication nonadherence and 26.9x more likely to diagnose DDIs compared to the control. Importantly, they also provided improved subsequent care: they were 3.3x more likely to restart the medication, the appropriate way to address nonadherence, and 15.7x more likely to stop or switch the interacting medications, the appropriate treatment for DDIs. The difference-in-difference calculations for DxTx scores, our combined measure of diagnostic and therapeutic improvement, confirmed this effect.

Although the CDM test could not explicitly test for disease progression, physicians could diagnose disease progression by deduction after the CDM test excluded NA and DDIs. Even though intervention physicians were more likely to identify disease progression, there was not a significant difference when compared to control in diagnosis or treating disease progression. Should future study confirm our findings that routine use of tools, such as the CDM test, can objectively exclude non-adherence and DDI, a clinician’s ability to determine whether worsening symptoms are due to worsening clinical conditions, ineffective medications, or misdiagnosis could be improved .

Interestingly, when we compared overall scores between the three case types, Afib, HF, and DM/HTN, we found no overall differences in diagnosis and treatment between each of the case types in aggregate. We interpret this finding as an indicator of the overriding challenge physicians face when caring for patients with multiple chronic conditions and accompanying polypharmacy, regardless of the specific disease, indicating the CDM Test has value across all four disease states.

Overall, only 1 in 8 providers in the elective intervention group chose to order the CDM test, suggesting that even with education, a more compelling narrative on these challenges than presented in our education materials is needed to alert physicians. Notwithstanding, Int2A were significantly more likely to make the primary diagnosis (58.1% vs. 16.5%) and order the correct related treatment (48.4% vs. 22.1%), compared to Int2B. These results closely mirrored the results we saw in the first intervention, who were all given the result, and control who were not.

The potential economic impact of the CDM test is compelling. Intervention ordered 0.3 fewer low-value diagnostic tests per case, leading to savings of $119. While savings realized through reduced utilization of low-value diagnostic testing is in line with the potential the CDM test costs, our analysis does not include larger direct costs; factoring in lower clinical, emergency, and hospital visits, the direct cost benefits would increase significantly. NA is estimated to cause 150,000 emergency room (ER) visits and over one million hospitalizations per year. DDIs, similarly, have been associated with 74,000 annual ER visits and 195,000 annual hospitalizations [23–25]. According to a study, the average cost of an ER visit is $383; reducing ED visits from NA and DDIs by 20% would compel over $17.0 million in savings from improved identification and treatment of NA and DDI using the CDM Test [26]. Hospital stays are estimated at $11,700/stay; if these hospitalizations are conservatively reduced by 10%, the CDM test would deliver a cost reduction of $1.2 billion for nonadherence and $228 million for DDIs [27–29].

Our findings have important implications for patients [28]. Similar to a previous study which only looked at DDIs in clinical practice, [29] these data show physicians are not checking for non-adherence and that the polypharmacy of chronic disease management is attended by unrecognized DDIs. When physicians are unable to distinguish between non-adherence and/or DDIs versus disease progression, assigning the correct treatment for these conditions becomes overwhelmingly more difficult [30]. After introducing an accurate, reliable, and standardized office-based test to identify medication nonadherence and DDIs among patients with chronic cardiometabolic conditions, we saw improvements not only in diagnostic accuracy but also, and perhaps more importantly, treatment for these common clinical conditions.

While we made a careful effort to present cases of chronic cardiometabolic conditions commonly encountered in primary care, the nine cases used in this study could not cover all possible presentations of medication adherence and DDIs. The CDM test does not test for every drug combination; and major drug groups used by these patients were not tested for, such as, parenteral medications (including insulin), but the test incorporates a significant number of commonly encountered prescription and non-prescription substances capable of contributing to pharmacokinetic or pharmacodynamic interactions when taken with prescription medications used to treat cardiometabolic disease. This study also did not collect patient-level data, and although CPV simulations have been validated against actual practice in numerous studies, future research can address this limitation [31]. Furthermore, the cases did not include patient specific factors that would lead to nonadherence or failure to recognize DDI such as language, literacy or cultural barriers. Finally, we did not perform a family-wise error reduction on our case variant analyses, as these were meant to show to the interested reader the effect of using the CDM test on each variant rather than as a primary outcome.

## Conclusion

Medication nonadherence and DDI are preventable sources of patient harm and poor health outcomes in chronic cardiometabolic disease management. Improved diagnosis using a reliable and convenient test potentially improves patient quality of life, medication safety, clinical outcomes, and cost-efficient health delivery.

## Electronic supplementary material

Below is the link to the electronic supplementary material.

Supplementary Material 1

Supplementary Material 2

## Data Availability

Data supporting these results available on reasonable request to the corresponding author.

## List of abbreviations

Afib: atrial fibrillation
AND: adherent, no drug-drug interaction
CDM: Chronic Disease Management
CPV: Clinical Performance and Value
DDI: drug-drug interaction
DM: diabetes mellitus
DxTx: diagnosis-treatment
HF: heart failure
HTN: hypertension
NA: non-adherence
O.R.: odds ratio
PCP: primary care physician
RCT: randomized controlled trial
