# Supplementary material for: Randomized prospective trial to detect and distinguish between medication nonadherence, drug-drug interactions, and disease progression in chronic cardiometabolic disease
Source: BMC Prim Care. 2023 Apr 15;24:100. doi: 10.1186/s12875-023-02042-4 (PMC10105436; doi:10.1186/s12875-023-02042-4)
Supplement: Supplementary file 1 — Supplementary Material 1 [file 12875_2023_2042_MOESM1_ESM.docx]

Supplement 1 – Pre-determined Case Scoring Criteria

| **HISTORY DOMAIN** |
| --- |
| Onset and duration of symptoms |
| Nature, progression and severity of symptoms |
| Associated symptoms |
| Previous workup/laboratory tests/imaging studies related to the complaint |
| Other medical problems and medical consults or hospitalizations |
| Past surgeries |
| Medications (prescription and over the counter) |
| Allergies to medications |
| Review of systems |
| History of notable diseases in family members |
| Alcohol, tobacco, illicit drug use |
| Lifestyle: diet, stressors, and physical activity |
| Social and economic determinants of health (employment status, insurance, living arrangement, relationship status) |
| **PHYSICAL EXAM DOMAIN** |
| General |
| HEENT/Head/Eyes/Ears/Nose/Throat |
| Neck |
| Lungs/Respiratory/Chest/Pulmonary |
| Cardiovascular/Cardiac/Heart/CV |
| Abdomen/Gastrointestinal/GI |
| Extremities/Musculoskeletal/MSK/Back |
| Dermatologic/Skin |
| Neurologic, as appropriate |
| Psychiatric/Depression Screening, as appropriate |
| **WORKUP DOMAIN** |
| Relevant ancillary tests (blood tests, urine tests, etc.) |
| Relevant imaging tests |
| Other applicable diagnostic procedures |
| **DIAGNOSIS DOMAIN** |
| Primary clinical diagnosis |
| Secondary diagnosis/-es |
| *Applicable to specific case type:* Medication non-adherence/ Drug-drug interaction |
| **TREATMENT DOMAIN** |
| *Applicable to specific case type:* Advise about importance of adherence, aids/tools to increase adherence, discontinue interacting medications, shift to different medication, increase medication dose, order interventions to address disease progression |
| Supportive treatment |
| Subspecialty referrals |
| Non-pharmacologic management |
| Follow-up office visit |
