# Supplementary material for: Randomized prospective trial to detect and distinguish between medication nonadherence, drug-drug interactions, and disease progression in chronic cardiometabolic disease
Source: BMC Prim Care. 2023 Apr 15;24:100. doi: 10.1186/s12875-023-02042-4 (PMC10105436; doi:10.1186/s12875-023-02042-4)
Supplement: Supplementary file 2 — Supplementary Material 2 [file 12875_2023_2042_MOESM2_ESM.docx]

Supplement 2 – CPV Patient Simulation Case Matrix Table

| **Case Types** | **Variant A:**  **CDM Test shows nonadherence but no DDI.** | **Variant B:**  **CDM Test shows DDI but not nonadherence.** | **Variant C:**  **CDM Test shows no nonadherence and no DDI.**  **Disease Progression** |
| --- | --- | --- | --- |
| **Atrial fibrillation (Afib)** | 77-year old woman reporting depressive symptoms while on follow-up for Afib (on warfarin), hypertension and dyslipidemia. Blood pressure, heart rate are controlled. INR is slightly below target. | 81-year-old woman on follow-up after recent transient ischemic attack. Known non-valvular Afib (on dabigatran), diabetes, dyslipidemia, and chronic low back pain. Recently started an OTC herbal supplement to help address symptoms of anxiety. | 66-year-old man on follow-up after recent cardioembolic stroke. Blood pressure controlled. TEE showed LA thrombus during stroke workup.  Known Afib (on apixaban), dyslipidemia, anxiety, and benign prostate enlargement. Recently started taking an OTC supplement for stress alleviation. |
| **Heart failure (HF)** | 67-year-old man with heart failure symptoms (orthopnea, easy fatigability) on maximal dose of beta blocker. Comorbid illnesses include HF with reduced ejection fraction, DM, chronic venous insufficiency, dyslipidemia, overweight, depression, chronic kidney disease stage 2. | 69-year-old man with known HF who presents with progressive symptoms (edema and easy fatigability) on optimal therapy. On history he has a recent urgent care visit for osteoarthritic knee pain, where he was prescribed celecoxib. He self-medicated with an unrecalled medication for knee pain as well. Comorbid illnesses include hypertension, depression, and benign prostatic hypertrophy. | 64-year-old woman with heart failure symptoms (orthopnea and nocturnal cough) on beta blocker and ARNI. Known HF with reduced ejection fraction, hypertension, and obesity. Recently diagnosed with depression and prescribed mirtazapine. Has also been taking NSAID sporadically for knee pain. |
| **Diabetes (DM)/ Hypertension (HTN)** | 78-year-old woman on follow-up, with poorly-controlled hypertension on telmisartan. On evaluation, patient also has mild depression.  Comorbid illnesses include, chronic kidney disease stage 3a, diabetes, anemia, dyslipidemia, osteoporosis, and osteoarthritis. | 61-year old man with low back pain and dizziness. Diagnosed with hypertension, diabetes, low back pain, dyslipidemia. Recently started an antibiotic for urinary tract infection. | 67-year-old woman complaining of headaches, with poorly controlled hypertension on maximum dose lisinopril/HCTZ. Has chronic Afib (on beta blocker and rivaroxaban) with controlled heart rate. Also diagnosed with diabetes, obesity, and chronic kidney disease stage 2. Currently taking an OTC supplement for wellness. |
